# Parental Acceptance of COVID-19 Vaccination for Children and Its Association With Information Sufficiency and Credibility in South Korea

**DOI:** 10.1001/jamanetworkopen.2022.46624

**Published:** 2022-12-14

**Authors:** Minjung Lee, Sujin Seo, Syngjoo Choi, Jung Hyun Park, Shinkyeong Kim, Young June Choe, Eun Hwa Choi, Geun-Yong Kwon, Jee Yeon Shin, Sang-Yoon Choi, Mi Jin Jeong, Hyunju Lee, Myoungsoon You

**Affiliations:** 1 Institute of Health and Environment, Seoul National University, Seoul, Republic of Korea; 2 Office of Dental Education, School of Dentistry, Seoul National University, Seoul, Republic of Korea; 3 Department of Public Health Sciences, Graduate School of Public Health, Seoul National University, Seoul, Republic of Korea; 4 Department of Economics, College of Social Sciences, Seoul National University, Seoul, Republic of Korea; 5 Department of Pediatrics, Korea University Anam Hospital, Seoul, Republic of Korea; 6 Department of Pediatrics, Seoul National University College of Medicine, Seoul, Republic of Korea; 7 Department of Pediatrics, Seoul National University Children’s Hospital, Seoul, Republic of Korea; 8 Division of Immunization, Korea Disease Control and Prevention Agency, Cheongju, Republic of Korea; 9 COVID-19 Vaccination Task Force, Korea Disease Control and Prevention Agency, Cheongju, Republic of Korea; 10 Department of Pediatrics, Seoul National University Bundang Hospital, Seongnam, Republic of Korea

## Abstract

**Importance:**

Vaccination against COVID-19 is an effective method for individuals to reduce negative health outcomes. However, widespread COVID-19 vaccination among children has been challenging owing to parental hesitancy.

**Objective:**

To examine parental decision-making in favor of the COVID-19 vaccine for their children and its association with the sufficiency and credibility of the information about the vaccine.

**Design, Setting, and Participants:**

This cross-sectional survey study was conducted in South Korea from February 7 to 10, 2022, 7 weeks before initiation of the COVID-19 vaccine for children aged 5 to 11 years. Parents were included if they spoke Korean and had at least 1 child in elementary school (grades 1-6). Parents and children were included in a 1:1 ratio; a total of 113 450 parents and 113 450 children were included in the analysis. Statistical analysis was performed between March and April 2022.

**Main Outcomes and Measures:**

The main outcomes of interest were (1) parental acceptance of COVID-19 vaccination for their children and (2) its association with self-reported sufficiency and credibility of information about the vaccine. A multivariable logistic regression was used to evaluate factors associated with parental decision-making in favor of COVID-19 vaccination; path analysis was used to examine indirect effects of information sufficiency and credibility.

**Results:**

Of the 113 450 children, 58 342 (51.4%) were boys, and the mean (SD) age was 10.1 (1.5) years. Of the 113 450 parents who responded, 7379 (6.5%) were accepting vaccination for their children; 15 731 (13.9%) reported the vaccine-related information they received was sufficient, and 23 021 (20.3%) reported the information was credible. Parents who reported that the information was sufficient were 3.08 times (95% CI, 2.85-3.33; *P* < .001) more likely to report being willing to vaccinate their children than those who believed the information was insufficient, and those who reported that the information was credible were 7.55 times (95% CI, 6.46-8.87; *P* < .001) more likely to report being willing to vaccinate their children than those who believed the information was not credible. Higher levels of information sufficiency and credibility were associated with perceptions of increased vaccine safety (sufficiency: β = 0.08; *P* < .001; credibility: β = 0.59; *P* < .001) and effectiveness (sufficiency: β = 0.05; *P* < .001; credibility: β = 0.60; *P* < .001).

**Conclusions and Relevance:**

In this study, a significant association was found between self-reported sufficiency and credibility of vaccine-related information and parental decision-making regarding COVID-19 vaccination for their children, suggesting that communications and policies that provide sound information are essential to improve vaccination rates.

## Key Points

**Question:**

What is the association of the sufficiency and credibility of information about COVID-19 vaccination for children with parental decisions regarding the vaccine?

**Findings:**

In this cross-sectional survey study of 113 450 parents, 6.5% were willing to vaccinate their child as soon as possible. Parents who perceived vaccination information to be sufficient were 3.08 times more likely to accept the vaccine than those who did not, and parents who perceived information to be credible were 7.55 times more likely to accept the vaccine than those who did not.

**Meaning:**

This study suggests that providing sufficient and credible information on COVID-19 vaccines may encourage parental decision-making in favor of vaccinating children.

## Teaser

This cross-sectional survey study examines parental decision-making in favor of the COVID-19 vaccine for their children and its association with the sufficiency and credibility of the information about the vaccine.

## Introduction

Vaccinating children against COVID-19 is recommended by pediatricians, medical experts, and health authorities.^1,2^ However, widespread COVID-19 vaccination among children has been challenging owing to a high rate of hesitancy among parents to vaccinate children.^3^ Although the hesitancy to vaccinate children and the antivaccination movements among parents have always been present,^4,5,6^ they are even more present because of the unfamiliarity with the novel COVID-19 vaccine.^7^ As data on short-term adverse effects have been reported,^7,8^ concerns regarding the short-term and long-term adverse effects have been raised.^8,9,10^

Decision-making on vaccination takes into account information regarding disease severity and susceptibility as well as the risks and benefits of the vaccine.^11^ Increased patient autonomy and active patient participation has led to deeper engagement of individuals in their own health.^12^ Roles and expectations regarding information have shifted, giving way to informed decision-making,^13,14^ which allows for choices reflecting the individual’s values^15^ to take priority, including those related to vaccination.^6,16^

In this cross-sectional survey study, we investigated how the sufficiency and credibility of information about the COVID-19 vaccine were associated with parental attitudes toward the vaccine for their children. Information sufficiency refers to an individual’s level of confidence in their knowledge of the potential risks in making their own health decisions^17,18^; information sufficiency is especially important for making decisions regarding vaccinations.^19^ When an individual feels inadequately informed, they may experience difficulty in taking action.^18^ Information insufficiency usually arises from either a lack of relevant information reflecting an individual’s specific circumstances or ineffective communication channels.^20^ Credibility of information is also an important factor in the public’s adherence to health recommendations.^21^ The credibility of information is associated with behavioral changes by altering perceptions and attitudes.^22,23^ During health crises, individuals’ trust in both the information and the communication channels delivering such information is critical for decision-making.^24,25^ For instance, a study reported that respondents who perceived that information from the Centers for Disease Control and Prevention was credible also perceived that the vaccine was safe.^26^

Studies have investigated the factors associated with parental decision-making in favor of vaccinating their children against COVID-19.^27,28,29,30^ However, there is limited knowledge on how information about the vaccine is associated with vaccination decisions. Because low-quality information can undermine an individual’s ability to make informed decisions and lead to harmful consequences,^31^ we focus on the association of information sufficiency and credibility with parental acceptance of COVID-19 vaccination for their children. This study aimed to (1) examine the level of parental COVID-19 vaccination acceptance for children in South Korea, (2) quantify and test the association of information sufficiency and credibility with decision-making in favor of COVID-19 vaccine acceptance, and (3) examine how attitudes toward COVID-19 vaccination were associated with information sufficiency and credibility.

## Methods

### Study Design and Participants

We conducted an online cross-sectional survey study from February 7 to 10, 2022, 7 weeks before COVID-19 vaccination was initiated for children aged 5 to 11 years in South Korea. This study was performed at a time during which the Omicron variant detection rate among confirmed South Korean cases of COVID-19 was more than 90%.^32^ An anonymous online questionnaire (eMethods in Supplement 1) consisting of 22 questions was developed to evaluate parental acceptance of COVID-19 vaccination for their children and assess the association between parental vaccine-related attitudes and perceptions of information about the COVID-19 vaccine. Study participants included parents of children in elementary school (grades 1-6) who were recruited nationwide via web-based notices, which are commonly used for school announcements. More than 113 510 participants were included in the survey; 113 450 participant responses were included in the analysis after excluding incomplete responses. The survey was exempted from review by the Seoul National University Bundang Hospital institutional review board because it was conducted as part of a public health prevention activity in collaboration with the Korea Disease Control and Prevention Agency and the Ministry of Education for the purpose of developing vaccination policies for children in Korea. The respondents provided electronic informed consent before completing the online self-reporting questionnaire. Responses from parents were collected voluntarily and anonymously; no personal data were obtained. The study followed the American Association for Public Opinion Research (AAPOR) reporting guideline for survey studies.

### Measures

A 5-point scale was used to measure parents’ intentions to vaccinate their child against COVID-19. Participants were asked, “Which of the following is close to your intention to vaccinate your child against COVID-19?” Participants could respond with the following options: “Willing to get my child vaccinated as soon as possible,” “Willing to get my child vaccinated but I want to wait and see,” “Not willing to get my child vaccinated but I want to wait and see,” “Not willing to get my child vaccinated at all,” and “Don’t know/not sure.” The option “Willing to get my child vaccinated as soon as possible” was coded as vaccine acceptance and assigned a value of 1, and all other options were coded as vaccine hesitancy and assigned a value of 0 to create a dichotomous acceptance vs hesitancy variable.

To assess for the sufficiency of vaccine-related information, the respondents were asked, “How confident are you that you have enough information to decide whether your child will be vaccinated against COVID-19?” In addition, to assess the credibility of vaccine-related information, we asked “Which of the following is the closest to the level of your agreement with respect to the credibility of COVID-19 vaccine information currently provided or accessed?” Responses were rated on a 5-point rating scale, with 1 indicating not sufficient or credible at all and 5 indicating very sufficient or credible. Additional survey items collected data including the child’s sex, school grade, and subjective health status. We also investigated the parents’ COVID-19 vaccination status (fully vaccinated, partially vaccinated, or not vaccinated at all).

Questions to assess parents’ attitudes that could be associated with COVID-19 vaccination were adapted from the Health Belief Model; these questions addressed the perceived risk of COVID-19 infection, the perceived benefits of COVID-19 vaccination, and the perceived barriers to vaccination.^33^ The perceived risk of COVID-19 infection comprised 2 components: perceived susceptibility and perceived severity.^34^ Respondents were asked, “What do you think is the probability that your child will be infected with COVID-19?” and “What do you think will be the severity if your child is infected with COVID-19?” Responses were rated on a 5-point scale, with 1 indicating very low, 2 indicating low, 3 indicating neither low nor high, 4 indicating high, and 5 indicating very high. The perceived benefits of COVID-19 vaccination were assessed by measuring the perceived effectiveness of preventing infection, severe infection, or death due to COVID-19. The perceived barriers to children receiving the COVID-19 vaccine were assessed by measuring the perceived safety of the vaccine. Responses for both of these questions were on a 4-point scale, with 1 indicating not at all and 4 indicating very much; responses for these questions included a “don’t know” option.

### Statistical Analysis

Statistical analysis was performed between March and April 2022. All quantitative variables were reported as frequencies, percentages, and mean (SD) values. Differences in study participant characteristics and information were compared with parental acceptance of the COVID-19 vaccination using χ^2^ statistics; *P* values are reported in Table 1.

**Table 1.** Characteristics Associated With Parental Intention to Vaccinate Children as Soon as Possible

Characteristic	All participants, No. (%) (N = 113 450)	Likelihood of getting the vaccine, No. (%)^a^		*P* value
		Acceptance (n = 7379)	Hesitancy (n = 106 071)	
**Children**				
Sex				
Male	58 342 (51.4)	3887 (6.7)	54 455 (93.3)	.03
Female	55 108 (48.6)	3492 (6.3)	51 616 (93.7)	
Grade				
1	3598 (3.2)	193 (5.4)	3405 (94.6)	<.001
2	19 315 (17.0)	683 (3.5)	18 632 (96.5)	
3	19 482 (17.2)	844 (4.3)	18 638 (95.7)	
4	22 718 (20.0)	1233 (5.4)	21 485 (94.6)	
5	23 917 (21.1)	1733 (7.2)	22 184 (92.8)	
6	24 420 (21.5)	2693 (11.0)	21 727 (89.0)	
Child’s subjective health				
Good	100 535 (88.6)	6953 (6.9)	93 582 (93.1)	<.001
Moderate	11 778 (10.4)	398 (3.4)	11 380 (96.6)	
Poor	1137 (1.0)	28 (2.5)	1109 (97.5)	
**Parents**				
Vaccination status				
Fully vaccinated	55 432 (48.9)	5994 (10.8)	49 438 (89.2)	<.001
Partially vaccinated	45 950 (40.5)	934 (2.0)	45 016 (98.0)	
Not vaccinated	12 068 (10.6)	451 (3.7)	11 617 (96.3)	
Information sufficiency				
Insufficient	49 935 (44.0)	1553 (3.1)	48 382 (96.9)	<.001
Moderate	47 784 (42.1)	3109 (6.5)	44 675 (93.5)	
Sufficient	15 731 (13.9)	2717 (17.3)	13 014 (82.7)	
Information credibility				
Not credible	41 467 (36.6)	211 (0.5)	41 256 (99.5)	<.001
Moderate	48 962 (43.2)	1482 (3.0)	47 480 (97.0)	
Credible	23 021 (20.3)	5686 (24.7)	17 335 (75.3)	
Perceived susceptibility of child to COVID-19 infection				
Low	26 845 (23.7)	1835 (6.8)	25 010 (93.2)	<.001
Moderate	64 550 (56.9)	3323 (5.1)	61 227 (94.9)	
High	22 055 (19.4)	2221 (10.1)	19 834 (89.9)	
Perceived severity of COVID-19 infection for child				
Low	15 894 (14.0)	802 (5.0)	15 092 (95.0)	<.001
Moderate	45 462 (40.1)	2523 (5.5)	42 939 (94.5)	
High	52 094 (45.9)	4054 (7.8)	48 040 (92.2)	
Perceived safety of COVID-19 vaccine				
Low	72 008 (63.5)	666 (0.9)	71 342 (99.1)	<.001
High	22 159 (19.5)	5869 (26.5)	16 290 (73.5)	
Don’t know	19 283 (17.0)	844 (4.4)	18 439 (95.6)	
Perceived effectiveness of COVID-19 vaccine				
Low	45 341 (40.0)	311 (0.7)	45 030 (99.3)	<.001
High	44 285 (39.0)	6391 (14.4)	37 894 (85.6)	
Don’t know	23 824 (21.0)	677 (2.8)	23 147 (97.2)	

^a^ Percentages are calculated from the row total.

A 2-stage multivariable analysis was performed. First, we performed logistic regression to evaluate factors associated with parental acceptance of the COVID-19 vaccination, including respondents’ characteristics as well as the perceived risk of infection, the benefits of and barriers to COVID-19 vaccination, and the sufficiency and credibility of information. Statistical analyses were conducted using R, version 4.2.1 (R Group for Statistical Computing)^35^; odds ratios (ORs), 95% CIs, and *P* values are reported. In the second stage, a mediation model was tested using path analysis in SPSS Amos, version 25 (SPSS Inc), with full information maximum likelihood estimation used to examine the association of information with parental acceptance of the COVID-19 vaccination as dependent variables.^36^ We controlled for all confounders and the potential association between participants’ characteristics and vaccine acceptance. In addition, the indirect effects of information sufficiency and credibility via mediators (ie, perceived risk of infection, safety of the vaccine, and effectiveness of the vaccine) were calculated using the PROCESS macro model 6 with 5000 bootstrap samples for SPSS, version 25 (IBM Corp). We used bootstrapped bias-corrected 95% CIs and *P* values to assess the significance of the standardized indirect associations. Indirect effects are assumed to be significant when the 95% bias-corrected CI does not cross zero. A 2-sided *P* < .05 was considered to be statistically significant.

## Results

### Characteristics of Participants

Parents and children were included in a 1:1 ratio. Of the 113 450 children, 58 342 (51.4%) were boys, and 55 108 (48.6%) were girls, with a mean (SD) age of 10.1 (1.5) years (Table 1). Respondents (N = 113 450) indicated that their children’s subjective health was good (100 535 [88.6%]), moderate (11 778 [10.4%]), and poor (1137 [1.0%]). Among the 113 450 parents, almost half reported they had received 3 doses of the COVID-19 vaccine (fully vaccinated; 55 432 [48.9%]), 45 950 (40.5%) reported they had received 1 to 2 doses of the vaccine, and 12 068 (10.6%) reported they had not been vaccinated.

### Parental Acceptance of COVID-19 Vaccination for Children

Of the 113 450 respondents, 7379 (6.5%) were willing to vaccinate their child as soon as possible, 32 739 (28.9%) were willing to vaccinate their child but wanted to wait and see, 26 030 (22.9%) were not willing to get their child vaccinated but wanted to wait and see for decision-making, and 43 174 (38.1%) were not willing to vaccinate their child at all. A total of 4128 parents (3.6%) marked “Don’t know/not sure” as the answer to the question about intention to vaccinate their child against COVID-19.

### Perceptions of the Sufficiency and Credibility of COVID-19 Vaccine Information

We examined the proportion of respondents who reported that vaccine-related information was sufficient and credible; only 15 731 (13.9%) reported that information was sufficient, while 49 935 (44.0%) reported that information was insufficient (Table 1). A total of 23 021 respondents (20.3%) reported that the vaccine-related information they received was credible; however, 41 467 (36.6%) reported that the information was not credible.

Differences in both child and parental factors were compared with parental acceptance of the vaccine using χ^2^ statistics. Parents were more likely to have favorable attitudes toward COVID-19 vaccination for a male child than for a female child (6.7% [3887 of 58 342] vs 6.3% [3492 of 55 108]; *P* = .03). In addition, respondents were significantly more likely to favor the vaccination if their child was in a higher grade (first grade, 5.4% [193 of 3598]; sixth grade, 11.0% [2693 of 24 420]; *P* < .001). Parents who assessed their child to have good subjective health were more likely to favor COVID-19 vaccination than those who assessed their child to have poor subjective health (6.9% [6953 of 100 535] vs 2.5% [28 of 1137]; *P* < .001).

### Parental Attitudes About COVID-19 Infection and Vaccination for Children

The mean (SD) score of perceived susceptibility to COVID-19 was close to “neither low nor high” (score = 3) (mean [SD] score, 2.92 [0.84]); 26 845 respondents (23.7%) reported their child’s perceived susceptibility as low, and more than half of respondents reported it as moderate (64 550 [56.9%]) (Table 1). The mean (SD) perceived severity score was higher than that for perceived susceptibility (mean [SD] score, 3.41 [0.92]). About half of respondents reported that the severity of COVID-19 infection would be high (52 094 [45.9%]), while only 15 894 (14.0%) reported it would be low.

Respondents’ attitudes about vaccination were assessed by 2 items that measured the perceived safety and effectiveness of COVID-19 vaccination. The mean (SD) perceived safety score was close to low (score = 2) (mean [SD] score, 1.98 [0.74]). Among the respondents, 72 008 (63.5%) reported the vaccine safety was low, and 22 159 (19.5%) reported the vaccine safety was high; 19 283 respondents (17.0%) reported that they did not know (Table 1). The mean (SD) score of the vaccine’s perceived effectiveness was close to low (mean [SD], 2.39 [0.82]). Among the respondents, 45 341 (40.0%) reported the effectiveness of the vaccine as low, while 44 285 (39.0%) reported it was high.

**Figure 1.**
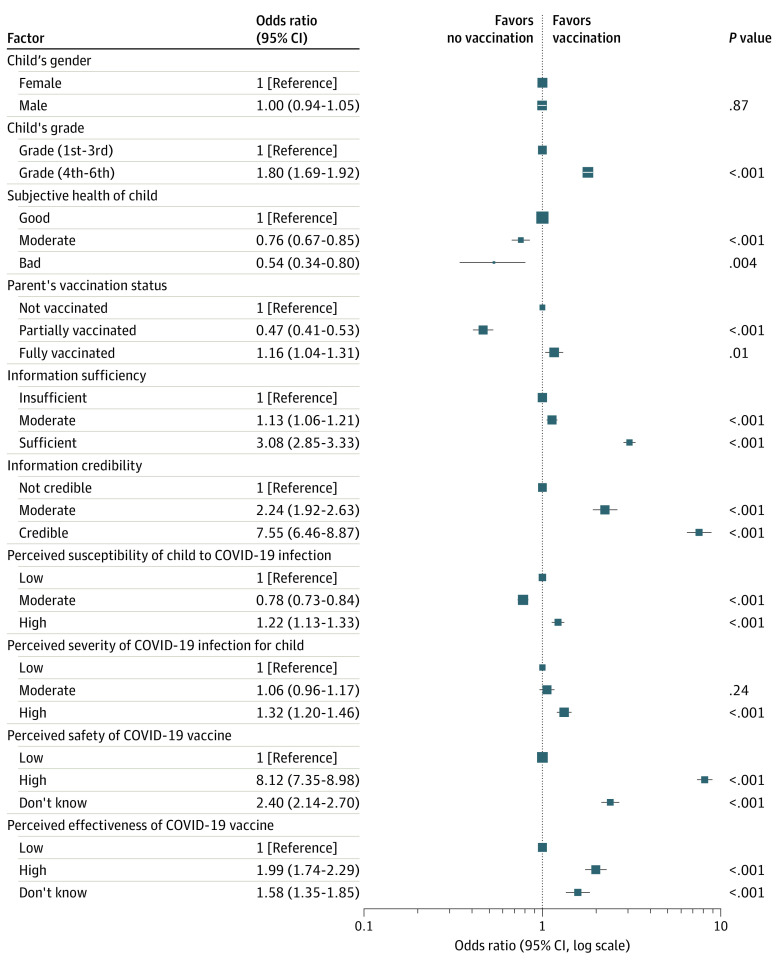
Forest Plot of Odds Ratios on Parental Intention to Vaccinate Children With the COVID-19 Vaccine

Table 2 and Figure 1 show results of the hierarchical logistic regression models used to test the association between vaccine acceptance and respondents’ characteristics, perception toward information, and attitude toward vaccine and infection;47.6% of the variance was explained by the final model. The perceived sufficiency and credibility of information were associated with higher rates of vaccine acceptance. Parents who perceived information to be sufficient (OR, 3.08 [95% CI, 2.85-3.33]; *P* < .001) and credible (OR, 7.55 [95% CI, 6.46-8.87]; *P* < .001) were more likely to accept child’s vaccination. High perceived susceptibility of their child to COVID-19 (OR, 1.22 [95% CI, 1.13-1.33]; *P* < .001) and high perceived severity of COVID-19 for their child (OR, 1.32 [95% CI, 1.20-1.46]; *P* < .001) were also associated with vaccine acceptance. In addition, perceived safety of the vaccine (OR, 8.12 [95% CI, 7.35-8.98]; *P* < .001) and perceived effectiveness of the vaccine (OR, 1.99 [95% CI, 1.74-2.29]; *P* < .001) were also associated with vaccine acceptance.

**Table 2.** Factors Associated With Parental Intention to Vaccinate Children as Soon as Possible

Variable	OR (95% CI)	*P* value
Child’s sex		
Female	1 [Reference]	NA
Male	1.00 (0.94-1.05)	.87
Child’s grade		
1-3	1 [Reference]	NA
4-6	1.80 (1.69-1.92)	<.001
Subjective health of child		
Good	1 [Reference]	NA
Moderate	0.76 (0.67-0.85)	<.001
Poor	0.54 (0.34-0.80)	.004
Parent’s vaccination status		
Not vaccinated	1 [Reference]	NA
Partially vaccinated	0.47 (0.41-0.53)	<.001
Fully vaccinated	1.16 (1.04-1.31)	.01
Information sufficiency		
Insufficient	1 [Reference]	NA
Moderate	1.13 (1.06-1.21)	<.001
Sufficient	3.08 (2.85-3.33)	<.001
Information credibility		
Not credible	1 [Reference]	NA
Moderate	2.24 (1.92-2.63)	<.001
Credible	7.55 (6.46-8.87)	<.001
Perceived susceptibility of child to COVID-19 infection		
Low	1 [Reference]	NA
Moderate	0.78 (0.73-0.84)	<.001
High	1.22 (1.13-1.33)	<.001
Perceived severity of COVID-19 infection for child		
Low	1 [Reference]	NA
Moderate	1.06 (0.96-1.17)	.24
High	1.32 (1.20-1.46)	<.001
Perceived safety of COVID-19 vaccine		
Low	1 [Reference]	NA
High	8.12 (7.35-8.98)	<.001
Don’t know	2.40 (2.14-2.70)	<.001
Perceived effectiveness of COVID-19 vaccine		
Low	1 [Reference]	NA
High	1.99 (1.74-2.29)	<.001
Don’t know	1.58 (1.35-1.85)	<.001

Abbreviations: NA, not applicable; OR, odds ratio.

**Figure 2.**
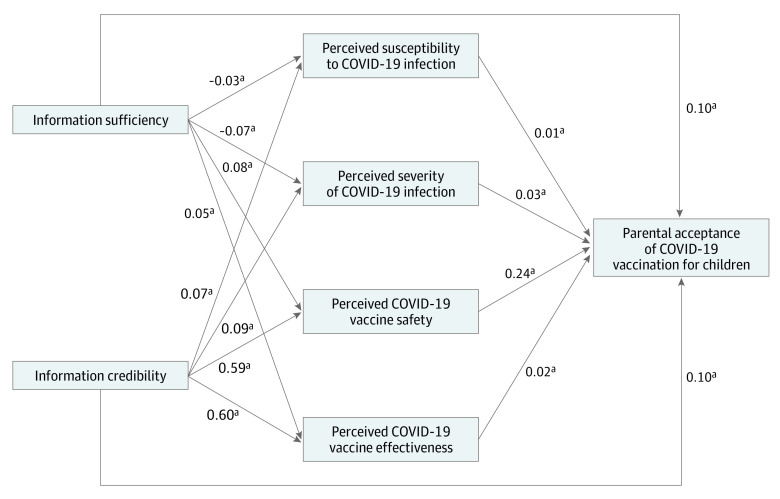
Path Model of Associations Between Information Perception, Attitudes Toward COVID-19 Infection and Vaccination, and Vaccine Acceptance ^a^*P* < .001.

Figure 2 presents the results of the path model analysis showing associations between perceptions of information, attitudes, and vaccine acceptance. Higher levels of information sufficiency were associated with lower perceived susceptibility of the child to COVID-19 (β = −0.03; *P* < .001), lower perceived severity of COVID-19 for the child (β = −0.07; *P* < .001), higher vaccine safety (β = 0.08; *P* < .001), and higher perceived effectiveness of the vaccine (β = 0.05; *P* < .001). Information credibility was associated with higher perceived susceptibility of the child to COVID-19 (β = 0.07; *P* < .001) and severity of COVID-19 for the child (β = 0.09; *P* < .001), as well as higher vaccine safety (β = 0.59; *P* < .001) and perceived effectiveness of the vaccine (β = 0.60; *P* < .001). We found that credible and sufficient information was indirectly associated with parental acceptance of the COVID-19 vaccine by altering perceptions regarding children’s susceptibility to COVID-19 infection and severity of COVID-19 infection for children as well as vaccine safety and effectiveness (Figure 2).

We tested associations of the sufficiency and credibility of information indirectly with parental vaccine acceptance via mediators (parental attitude toward vaccination). Most of the indirect effects tested were found to be significant, except for perceived susceptibility (Table 3). Information credibility via perceived vaccine safety was found to be the most robust (estimate, 0.587 [95% CI, 0.559-0.616]).

**Table 3.** Bootstrapped Bias-Corrected Estimates for the Indirect Effects of Information Sufficiency and Credibility on Parental Vaccine Acceptance via Mediators

Independent variable and mediator	Estimate (95% CI)
Information sufficiency	
Perceived susceptibility of child to COVID-19 infection	0.000 (−0.001 to 0.000)
Perceived severity of COVID-19 infection for child	−0.008 (−0.010 to −0.006)
Perceived effectiveness of COVID-19 vaccine	0.091 (0.083 to 0.098)
Perceived safety of COVID-19 vaccine	0.210 (0.200 to 0.221)
Information credibility	
Perceived susceptibility of child to COVID-19 infection	0.001 (−0.001 to 0.003)
Perceived severity of COVID-19 infection for child	0.016 (0.013 to 0.019)
Perceived effectiveness of COVID-19 vaccine	0.232 (0.205 to 0.262)
Perceived safety of COVID-19 vaccine	0.587 (0.559 to 0.616)

## Discussion

This study revealed a high level of COVID-19 vaccine hesitancy among parents of elementary school students in South Korea. Among the respondents, only 6.5% reported that they were willing to get their child vaccinated as soon as possible, 28.9% were willing to vaccinate their child but wanted to wait and see, 22.9% were not willing to get their child vaccinated but wanted to wait and see, and 38.1% were not willing to get their child vaccinated at all. The proportion of parents willing to vaccinate their child in our sample was lower than the threshold for COVID-19 herd immunity, which is estimated to be between 55% and 82% of the population, including children.^37^ This study demonstrates the need for further efforts to enhance parental acceptance of the COVID-19 vaccine for their children.

The results of this study also suggest that perceptions regarding information have a significant and robust association with parental decision-making in favor of vaccinating their children. Respondents who perceived the information to be sufficient were 3.08 times more likely to vaccinate their children than those who judged the information to be insufficient. In agreement with previous studies,^38,39^ the results of this study indicated that perceived sufficiency of information was positively associated with vaccine acceptance as well as perceived vaccine safety and effectiveness.

Respondents who perceived vaccine information to be credible were 7.55 times more likely to favor vaccinating their child than those who did not perceive information to be credible; however, only 20.3% of respondents reported that the vaccine-related information they received was credible. Perceived credibility of information was associated with vaccine acceptance indirectly by altering perceptions and attitudes regarding vaccine safety and effectiveness.^22,23^ Previous studies have shown that people adhere to health recommendations, including vaccination campaigns, only when they perceived the information to be credible.^21,40^ However, the “infodemics” phenomenon (the rapid spread and amplification of vast amounts of valid and invalid information in the media) remains an obstacle to combating the COVID-19 pandemic.^41^ People perceive information as more credible when it fits with existing beliefs and is obtained from credible sources.^42^ Parental hesitancy regarding decision-making for their child’s vaccination may be associated with receiving conflicting information from a range of sources.

We found that parents’ hesitation was mainly due to their lack of confidence in the vaccine’s effectiveness and safety.^43,44^ Previous studies also showed that doubts about the safety of vaccines hindered caregivers’ decisions to have their children receive not only routinely administered vaccines^45^ but also the COVID-19 vaccine.^27,46^ This study revealed that information sufficiency and credibility are highly associated with confidence in the COVID-19 vaccine. Causal models of relevant studies propose that knowledge heightened by information influences behavioral change indirectly by altering perceptions of risks and benefits.^47,48,49^ Therefore, the provision of information about vaccine safety issues and efficacy can have a positive association with attitudes toward vaccines and, consequently, vaccine uptake at the population level.

A total of 28.9% and 22.9% of the respondents were willing and not willing to get their child vaccinated but wanted to wait and see, respectively. From a public health perspective, this group of individuals is an important target for outreach because converting them from hesitancy to acceptance is relatively easier than converting those in the group of respondents who were not willing to vaccinate their child at all. Also, pediatric physicians may play a crucial frontline role in encouraging COVID-19 vaccination during physician-patient encounters. Physicians are reportedly the most trusted source of information and influence decision-making regarding vaccination.^3,50,51^ Moreover, physicians can provide information reflecting the consideration of patients’ specific circumstances. Thus, recommendations by physicians can bolster support for the COVID-19 vaccine. Last, negative information tends to influence evaluations more strongly than comparably positive information.^52,53^ Although social media are powerful tools for disseminating evidence-based health information and recommendations to large numbers of people, there are concerns about inaccurate data, rumors, and misinformation on these platforms.^54,55^ This suggests that providing positive messages about vaccination can be an effective intervention.

### Limitations

This was a large-scale study, with a nationwide sample and statistical power; however, it has several limitations. This study focused on the association between parents’ perceptions of vaccine-related information and vaccine acceptance. However, questions regarding how much information is perceived to be sufficient, factors associated with information sufficiency (ie, the amount of information provided and the information delivered to parents), and specific information associated with parental decisions were not investigated in this study. Future studies focusing on information sufficiency, including qualitative aspects of vaccine-related information, are needed. Moreover, variables such as parents’ socioeconomic backgrounds, spousal agreement, and previous experience with vaccines were not evaluated. Another factor not analyzed in this study that may influence parental acceptance of the vaccine was whether a child had previously contracted SARS-CoV-2. As of February 10, 2022, among South Korean children aged 5 to 11 years, 103 145 COVID-19 cases were diagnosed, which accounts for 3.3% of the total population of this age group. Therefore, most children were COVID-19 naive at the time of the survey.^56^ Last, parents provided responses during a time of intense uncertainty, with major changes in daily activities (eg, no school and work at home); responses may differ in different social settings or if the number of cases increases.

## Conclusions

In this study, only 6.5% of parents with children in elementary school were accepting of COVID-19 vaccination for their children. Perceptions regarding the sufficiency and credibility of vaccine-related information had a significant and robust association both directly and indirectly with parental decision-making regarding vaccinating their children. Therefore, the results of this study suggest that disseminating credible information about the safety and effectiveness of vaccines may be key to increasing the number of parents who are willing to vaccinate their children.

## Notes

Supplement 1.
eMethods.

Supplement 2.
Data Sharing Statement
