# Supplementary material for: Parental Acceptance of COVID-19 Vaccination for Children and Its Association With Information Sufficiency and Credibility in South Korea
Source: JAMA Netw Open. 2022 Dec 14;5(12):e2246624. doi: 10.1001/jamanetworkopen.2022.46624 (PMC9856328; doi:10.1001/jamanetworkopen.2022.46624)
Supplement: Supplement 1. — eMethods. [file jamanetwopen-e2246624-s001.pdf]

Supplementary Online Content

Lee M, Seo S, Choi S, et al. Parental acceptance of COVID-19 vaccination for children and its association with information sufficiency and credibility in South Korea. *JAMA Netw Open.* 2022;5(12):e2246624. doi:10.1001/jamanetworkopen.2022.46624

eMethods.

This supplementary material has been provided by the authors to give readers additional information about their work.

© 2022 Lee M et al. *JAMA Network Open.*

eMethods.

The information described below provides the questionnaire which was developed for this study.

1. How many children do you have in total?

1) 1 child 2) 2 children 3) 3 children 4) 4 or more children

Note. If you have more than two children, please think of one child in elementary school 1st to 6th and answer all the questions below.

2. Please select your child's birth year.

1) 2015 2) 2014 3) 2013 4) 2012 5) 2011 6) 2010 7) 2009 8) others

3. What grade will your child enter in March this year?

1) Grade1 2) Grade2 3) Grade3 4) Grade4 5) Grade5 6) Grade6

4. What is your child's gender?

1) Female 2) Male

5. In which of the following areas is your child's school?

1) Seoul 2) Busan 3) Daegu 4) Incheon 5) Gwangju 6) Daejeon 7) Ulsan 8) Sejong 9) Gyeonggi 10) Gangwon 11) Chungcheongbuk-do 12) Chungcheongnam-do 13) Jeollabuk-do 14) Jeollanam-do 15) Gyeongsangbuk-do 16) Gyeongsangnam-do 17) Jeju

6. How is your child's health?

1) Very good

2) Good

3) Normal

4) Not good

5) Very bad

© 2022 Lee M et al. *JAMA Network Open.*

7. Has your child been vaccinated over the past five years? (e.g., measles/spectrum/fungus (MMR), diphtheria/tide/whitening (DTP), Japanese encephalitis, flu, etc.)

1) Yes 2) No 3) I do not know/I do not remember

8. Which of the following is the case for you (parents) regarding COVID-19 vaccination?

1) fully vaccinated
2) partially vaccinated
3) not vaccinated at all

9. What do you think is the probability that your child will be infected with COVID-19?

1) Very low
2) Low
3) Neither low nor high
4) High
5) Very high

10. What do you think will be the severity if your child is infected with COVID-19?

1) Very low
2) Low
3) Neither low nor high
4) High
5) Very high

11. How confident are you that you have enough information to decide whether your child will be vaccinated against COVID-19?

1) Not sufficient at all
2) Not sufficient
3) Moderate

4) Sufficient

5) Very sufficient

12. Which of the following is close to your intention to vaccinate your child against COVID-19?

1) Willing to get my child vaccinated as soon as possible

2) Willing to get my child vaccinated but I want to wait and see

3) Not willing to get my child vaccinated but I want to wait and see

4) Not willing to get my child vaccinated at all

5) Don’t know/not sure

13. What do you think about the safety of the COVID-19 vaccine?

1) Not at all

2) a little

3) rather

4) Very much

5) Don’t know

14. What do you think about the effectiveness of the COVID-19 vaccine to prevent infection and prevent severe deaths?

1) Not at all

2) a little

3) rather

4) Very much

5) Don’t know

15. If you were to compare the benefits and risks of getting a COVID-19 vaccine for your child's health or daily life, which of the following do you think is closer?

1) Risk is much greater than the benefit

© 2022 Lee M et al. *JAMA Network Open.*

2) Risk is greater than the benefit

3) Risks and benefits are half and half

4) Benefits are greater than the risks

5) Benefits are much greater than the risks

6) I do not know

16. If you compare the risk of COVID-19 infection with the risk of getting vaccinated for the health and daily life of children aged 5-11, which of the following do you think is closer?

1) For children of this age, the risk of COVID-19 infection is much greater than the risk of vaccination.

2) For children of this age, the risk of COVID-19 infection is greater than the risk of vaccination

3) The risk of COVID-19 infection in children of this age is the same as the risk of being vaccinated.

4) For children of this age, the risk of vaccination is greater than the risk of COVID-19 infection

5) For children of this age, the risk of vaccination is much greater than the risk of COVID-19 infection

6) I do not know

17. If you recommend your child to be vaccinated, choose the two most important reasons.

1) To prevent my child from being infected and to prevent serious infections and complications caused by infection

2) To prevent my child from spreading the infection to family or people around him/her.

3) To let my child get back to his/her pre-COVID-19 life by getting vaccinated

4) In order for our society to recover daily life such as economic recovery

5) To avoid the inconvenience of quarantine activities (body temperature measurement, meal partition, mask use, etc.)

© 2022 Lee M et al. *JAMA Network Open.*

6) Not applicable

18. If you do not recommend your child to be vaccinated, choose the two most important reasons.

1) Don't want to give my child an injection

2) To avoid my child experiencing adverse reactions or side effects after vaccination

3) To avoid disturbing my child's daily life and study, concerning about vaccination

4) Don't think my child will get infected even if he/she doesn't get vaccinated.

5) The quarantine activities (body temperature measurement, meal partition, mask use, etc.) are enough, so don't want my child to get vaccinated.

6) Not applicable

19. Which of the following is the closest to the level of your agreement with respect to the credibility of COVID-19 vaccine information currently provided or accessed?

1) Not credible at all
2) Not credible
3) Moderate
4) Credible
5) Very credible

20. What is your opinion on COVID-19 vaccination?

1) Vaccination is for me

2) Vaccination is for the social community

3) Both

4) Neither

5) Don't know

21. Mark all relevant experiences related to COVID-19.

© 2022 Lee M et al. *JAMA Network Open.*

1) Me/my family (parents, siblings, grandparents, etc.) have been infected

2) Me/my family (parents, siblings, grandparents, etc.) have been quarantined as a close contact

3) My child's school (or academy) teacher or friend has been infected

4) My child's school (or academy) teacher or friend has been quarantined as a close contact

5) Not applicable

22. How safe do you think your child's school is from COVID-19?

1) Not very safe

2) Not safe

3) Normal

4) Safe

5) Very safe

© 2022 Lee M et al. *JAMA Network Open.*
