# Supplementary material for: Parental Acceptance of COVID-19 Vaccination for Children and Its Association With Information Sufficiency and Credibility in South Korea
Source: JAMA Netw Open. 2022 Dec 14;5(12):e2246624. doi: 10.1001/jamanetworkopen.2022.46624 (PMC9856328; doi:10.1001/jamanetworkopen.2022.46624)
Supplement: Supplement 2. — Data Sharing Statement [file jamanetwopen-e2246624-s002.pdf]

Data Sharing Statement

Lee. Parental Acceptance of COVID-19 Vaccination for Children and Its Association With Information Sufficiency and Credibility in South Korea. *JAMA Netw Open*. Published December 14, 2022. doi:10.1001/jamanetworkopen.2022.46624

Data
Data available: No
